# The Influence of Light Wavelength on Human HPA Axis Rhythms: A Systematic Review

**DOI:** 10.3390/life13101968

**Published:** 2023-09-26

**Authors:** Isabella Robertson-Dixon, Melanie J. Murphy, Sheila G. Crewther, Nina Riddell

**Affiliations:** 1 Department of Psychology, Counselling and Therapy, La Trobe University, Melbourne, VIC 3086, Australia; i.robertson-dixon@latrobe.edu.au (I.R.-D.); m.murphy@latrobe.edu.au (M.J.M.); s.crewther@latrobe.edu.au (S.G.C.); 2 Centre for Mental Health and Brain Sciences, Swinburne University of Technology, Melbourne, VIC 3122, Australia

**Keywords:** HPA axis, hypothalamic pituitary adrenal axis, stress, cortisol, wavelength, spectrum, circadian rhythm

## Abstract

Environmental light entrains many physiological and behavioural processes to the 24 h solar cycle. Such light-driven circadian rhythms are centrally controlled by the suprachiasmatic nucleus (SCN), which receives information from the short-wavelength-sensitive intrinsically photosensitive retinal ganglion cells. The SCN synchronizes local clocks throughout the body affecting sleep/wake routines and the secretion of neuroendocrine-linked hormones such as melatonin from the pineal gland and cortisol via the hypothalamic pituitary adrenal (HPA) axis. Although the effects of light parameters on melatonin have been recently reviewed, whether the experimental variation of the spectral power distribution and intensity of light can induce changes in cortisol rhythms remains unclear. Thus, this systematic review evaluated the effects of daytime exposure to lights of different spectral wavelength characteristics and luminance intensity on the cortisol levels in healthy individuals. A search of the PubMed, Web of Science, EMBASE, CINAHL, Medline, PsycINFO and Cochrane Library databases on 19 June 2023 identified 3418 articles, of which 12 studies (profiling 337 participants) met the inclusion and risk of bias criteria. An analysis of the literature indicated that exposure to bright lights of any colour during the late night or early morning can induce significant increases in cortisol secretion relative to time-matched dim light comparison conditions. Furthermore, exposure to bright lights with stronger short-wavelength (blue/green) components in the early morning typically induced greater increases in cortisol relative to lights with stronger long-wavelength (red) components. Thus, the circadian regulation of cortisol is sensitive to the wavelength composition of environmental lighting, in line with the more commonly studied melatonin. As such, wavelength characteristics should be optimized and reported in light intervention studies (particularly for the investigation of cortisol-associated disorders and HPA axis function), and exposure to short-wavelength light during sensitive periods should be carefully considered in constructed environments (e.g., bedroom and classroom lighting and device screens).

## 1. Introduction

Many animal species, including humans, are diurnal, being awake, active and eating during daylight hours and sleeping during darkness. Such environmentally determined circadian behaviours are typically associated with the 24 h cycling of neurological, endocrine, metabolic, cardiovascular, immune and behavioural functions. Hence, even short-term acute changes in the light experience of humans, such as during international air travel, can disrupt the 24 h circadian cycle of physiological and behavioural processes, including hormonal secretions of melatonin and the glucocorticoid stress hormone, cortisol [1].

Cortisol secretion is controlled by an interaction between the hypothalamic pituitary adrenal (HPA) axis and the light-driven suprachiasmatic nucleus (SCN), the master circadian pacemaker of the central nervous system [2]. SCN activity also facilitates an entrainment of feeding routines and of circadian clocks in peripheral tissues [3,4], and thus has wide-ranging effects on general health and mood [5,6]. Unlike melatonin, which is currently the primary nutraceutical rehabilitation focus for sleep anomalies [7], it is unclear if and how different spectral wavelengths of light impact cortisol secretion in healthy neurotypical adults at different times of the day/night cycle [8,9,10], such as the highest levels of the cortisol awakening response (CAR) and rate of fall off during the day.

Despite this, interventions involving exposure to lights with different spectral wavelength power distributions are being trialled for a variety of educational environments [11] and clinical conditions (e.g., seasonal affective disorder, depression and sleep disorders [12,13]). Moreover, commercially available products that alter the spectral characteristics of light in the environment are increasingly available (e.g., blue light digital screen filters [14] and tinted glasses for dyslexia and childhood migraines [15,16,17]). Thus, this review aimed to systematically evaluate the literature examining the effect of different spectral wavelengths of light on cortisol levels (in conjunction with melatonin levels when provided) at various times of the normal circadian day in humans.

### 1.1. The Circadian System, Cortisol and Melatonin

**Figure 1:**
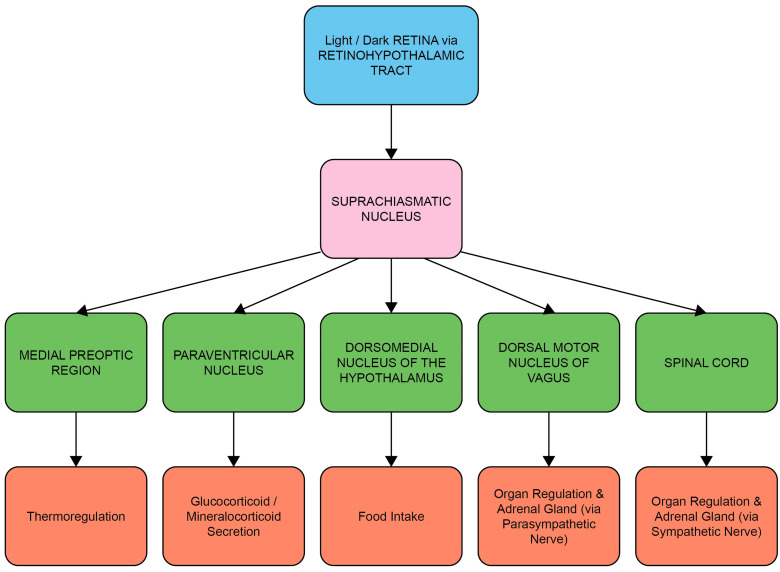
The circadian system: central and peripheral system communication through neural and hormonal interactions. Luminance information from the ipRGCs of the retina in the eye is passed to the suprachiasmatic nucleus (SCN) through the retino-hypothalamic tract (RHT), which differentially influences the synchronization of circadian rhythms throughout the body at different times of day. Neurons transmit this timing information to other parts of the brain and the intrinsic circadian clocks of peripheral organs, thus influencing activities such as hormone secretion, food intake, sleep and regulation of body temperature.

**Figure 2:**
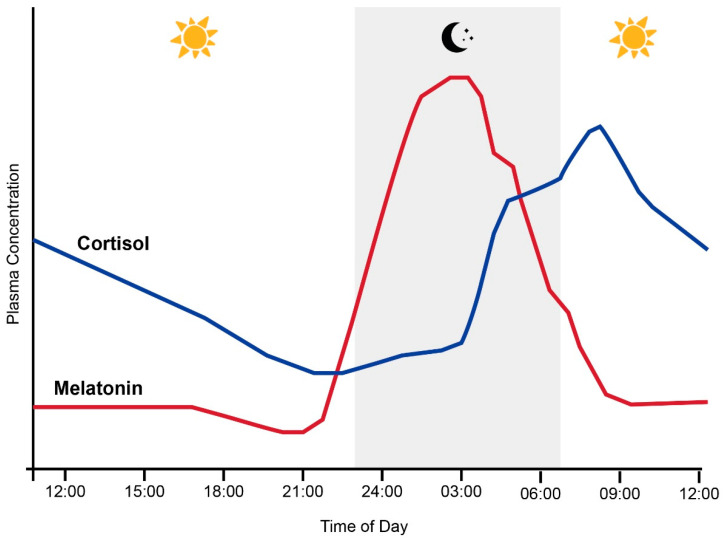
Normal relationship between plasma melatonin and cortisol levels and sleep (grey shading) in humans across the 24 h day. Adapted from Hickie et al. [27].

Entrainment of the circadian system is achieved by the transduction and transmission of information about daylight to the brain via the retinal photoreceptors (rods and cones) and melanopsin-containing intrinsically photosensitive retinal ganglion cells (ipRGCs) [18]. The ipRGCs in particular project via the retino-hypothalamic tract to the SCN, the synchronizing structure of the central circadian clock [19]. Light-induced activation of the SCN coordinates the circadian activity of other tissues in the brain and peripheral organs via neuronal and hormonal signals, such as rhythms in the secretion of melatonin from the pineal gland and cortisol via the adrenal cortex of the HPA axis [2,20] (Figure 1 and Figure 2). There is a strong bi-directional relationship between the HPA axis and the circadian system, with the SCN regulating HPA axis activity at multiple levels, including via neuronal connections to the paraventricular nucleus of the hypothalamus, to influence the rhythmic secretion of glucocorticoids [21].

Melatonin synthesis in the pineal gland is also controlled by the SCN through a multi-synaptic pathway in response to the environmental light/dark cycle. As illustrated in Figure 2, melatonin levels in humans with an intermediate chronotype typically slowly increase throughout the evening and peak late at night [22]. Conversely, the secretion of the glucocorticoid hormone cortisol from the adrenal glands typically peaks in the early morning and slowly declines throughout the day until approximately midnight [23]. This pattern is reinforced by the cortisol awakening response (CAR), a peak in cortisol levels typically occurring 30 to 40 min after waking [24]. Together with melatonin, this circadian rhythm in circulating cortisol provides a major endocrine signal to synchronize peripheral tissues to the 24 h clock [25]. However, it must be noted that the timing of these rhythms can shift depending on an individual’s circadian clock phase [26].

### 1.2. Entrainment of the Circadian System and HPA Axis by Light

The phase (timing) of peaks and troughs in SCN-controlled endogenous circadian rhythms, frequently assessed by measuring melatonin levels, can be changed by altering environmental light exposures. Indeed, the timing of light exposure determines the direction of the effect, with phase delays (shifts to a later time) occurring following light exposures during the early biological night, while phase advances (shifts to an earlier time) can be induced by late night/early morning light exposures [28]. This is experienced in the exposure to light shift in daylight savings in the summer photoperiod compared to the winter photoperiod, where the advancement of the circadian rhythm clock creates a phase shift in the morning melatonin decline and cortisol rise [29]. This shift is associated with the vasopressin-immunoreactive neurons in the SCN, whose overall volume fluctuates throughout the year depending on the season [30]. The ability of exogenous light to induce a phase shift can also depend on its intensity, duration and spectral composition [31]. Although intense light is relatively more effective in inducing phase shifts, the human circadian system can be highly sensitive to any light exposure, particularly in the evening when phase shifts, as indicated by plasma melatonin levels, can be shifted using very dim room lighting [32], though there is a large difference in the degree of sensitivity to evening light across individuals [33]. Thus, low light intensities can sometimes be adequate to induce a phase shift, while in other individuals, higher light intensities are needed to produce the same effect. With regards to wavelength, the sunlight that most primates are exposed to during the daylight cycle in their natural environment contains a full spectrum of visible light wavelengths ranging from ~400 to 700 nanometers (nm), which is perceived in colours ranging from blue to red [34]. In the retina, the ipRGCs that mediate circadian entrainment are hypersensitive to short-wavelength blue light (with a peak sensitivity of 480 nm) [18,35]. Consequently, blue light can be more effective than longer wavelengths in altering melatonin secretion (a traditional marker of circadian activity in response to darkness), depending on the intensity and duration of exposure [36,37,38].

While the effects of light exposure parameters on melatonin levels and sleep behaviours have been systematically reviewed previously [28,39]), the impact on circulating cortisol (a key effector of circadian rhythms in peripheral tissues [3]) remains relatively less understood. Most notably, although it is accepted that bright light is more effective than dim light in altering cortisol secretions, it remains unclear whether light-induced changes in cortisol secretion are also dependent on the light spectrum, with some studies reporting a cortisol spectrum effect [10,40,41,42] and others reporting spectrally dependent shifts in melatonin but not cortisol [43,44,45,46]. The light exposures tested in these studies included a range of combinations of circadian timing, duration, intensity and spectral distribution, presumably contributing to the different responses measured. Thus, work is now needed to systemically review the literature examining how the spectral distribution of light affects cortisol response, as well as how spectral effects are mediated by other exposure characteristics such as the circadian timing and intensity of light.

### 1.3. Systematic Review Aims

It is unclear how the spectral characteristics of light exposures affect the circadian rhythmicity of cortisol. Such knowledge is clinically important, with light intervention treatments increasingly being trialled for affective and behavioural disorders, such as depression [12], sleep disorders [13], and cancer-related fatigue [47,48], in which cortisol rhythms may play a role in the underlying pathophysiology [49,50,51,52]. Such interventions typically involve short daytime exposures to bright white or blue light; however, the spectral power distributions of the light sources are often not explicitly reported or optimized (see review by van Maanen et al. [53]), and an uncertainty about the baseline effects of different light spectrums on cortisol has complicated the interpretation of trial outcomes (e.g., [47]). Hence, following PRISMA guidelines, this paper aimed to systematically review all the available research articles examining the effects of exposure to light with different spectral characteristics during the typical photoperiod (i.e., awake time) on systemic biochemical measures of the HPA axis function in healthy human participants.

## 2. Methods

### 2.1. Search Strategy

This study was conducted in accordance with PRISMA guidelines [54] (see Supplementary Materials for PRISMA-P checklist) and registered with Open Science Framework (osf.io/h9arw (accessed on 7 July 2022). PubMed, Web of Science, EMBASE (Ovid), CINAHL, Medline (Ovid), PsycINFO and Cochrane Library databases were searched for peer-reviewed full-text studies written in the English language with no date restrictions. This combination of databases has been shown to provide optimal coverage of the literature [55] and includes additional databases with specialized foci that are relevant to this review. The search strategy illustrated in Table 1 was performed in PubMed and adapted for other databases. Reference lists of extracted studies were also manually searched. The final database search was run on 19 June 2023. Results were imported into Covidence Systematic Review Software (Veritas Health Innovation, 2022).

### 2.2. Study Selection and Risk of Bias Assessment

Studies were eligible for inclusion if they investigated how exposure to light of different wavelengths during the typical photoperiod (i.e., awake time) affected systemic biochemical measures of HPA axis function in healthy human participants. Studies were excluded if they investigated wavelengths that were not in the visible light range, if wavelength manipulations were only applied outside the participant’s usual photoperiod (e.g., assessment of blue light exposure during habitual sleep times), if participants had underlying pathology or deficits (e.g., cancer treatment or sleep restriction studies) without a health control comparator or baseline or if studies only assessed HPA axis function in non-systemic samples (e.g., in vitro studies or skin samples).

Study selection and data extraction were performed independently by two reviewers (IRD and NR). In Covidence, the reviewers first independently screened the title and abstract of each identified record and subsequently performed full text screening to determine whether to accept or reject a particular study based on the pre-specified inclusion/exclusion criteria. The decision to exclude a study was recorded using a hierarchy of eight exclusion reasons (Table 2). A quality assessment of the included studies was conducted using the OHAT Risk of Bias Rating Tool for Human and Animals studies (“OHAT risk of bias rating tool for human and animal studies”, 2015). Questions relevant only to animal studies, cohort, case-control, cross-sectional and case reports were excluded from the assessment, as outlined in the rating tool guidelines. Included questions are listed in Table 3.

### 2.3. Data Analysis

OHAT risk of bias assessments were conducted to determine the quality of each included study. Following this, data extraction was performed (Table 4). Where descriptive statistics for cortisol measurements were not available in the manuscript text or tables, the information was extracted from figures using Engauge Digitizer (v12.1) [58]. To enable comparison between the results of each study, extracted cortisol data were converted to percentage difference measures between conditions. Figures depicting cortisol difference measures were created using the web app Flourish [59] and Adobe Illustrator 2022 (v26.3.1). As most studies provided multiple cortisol measurements across brief time increments post-exposure, raw data were averaged to provide a summary measure for each 2 h time window post-exposure in visual diagrams. Similarly, where >2 h of pre-exposure baseline data were available, only the two hours immediately preceding the light exposure were extracted and averaged for visual display. For one study [45], where descriptive statistics were only available for the average of three time-points, the data are presented at the midpoint of the three timepoints in our figures. Two studies [40,46] provided insufficient descriptive statistics to enable a visual representation of their cortisol results, and thus their findings are described only qualitatively.

## 3. Results

### 3.1. Study Selection

**Figure 3:**
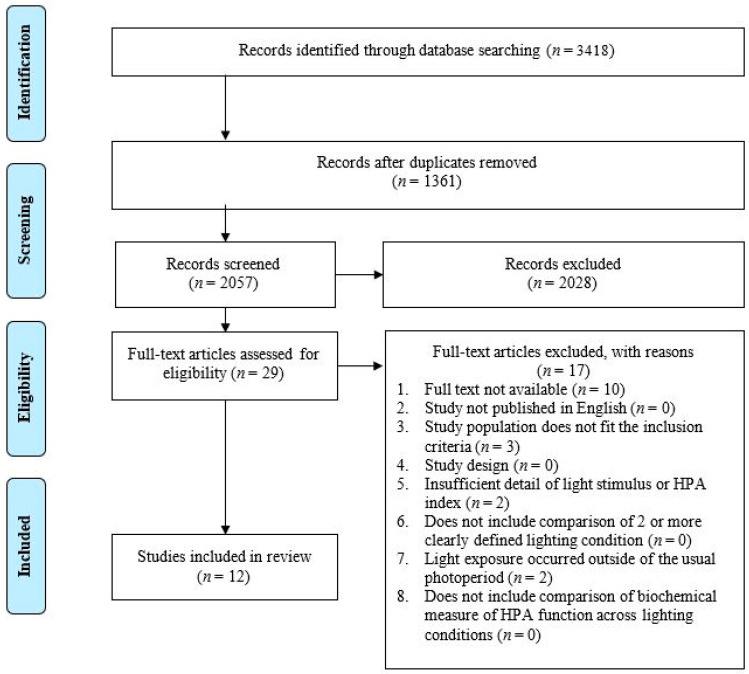
PRISMA flow diagram of the study selection process.

The database searching identified a total of 3418 citations. After the removal of 1361 duplicates, 2057 articles underwent title and abstract screening. Following the title and abstract screening, twenty-nine citations were identified as eligible for a full text review. Seventeen further articles were excluded during the full text review for not meeting all the inclusion criteria. Of these 17, 10 studies were excluded because a full text was not available [60,61,62,63,64,65,66], 3 did not include a population that met the inclusion criteria [67,68,69], 2 did not include a light exposure that occurred within the usual photoperiod (characterized as the participants habitual non-sleep times) [46,70], and 2 did not include sufficient detail with regard to the description of the light stimulus [71,72]. A total of twelve articles were identified for inclusion in the systematic review (Figure 3).

### 3.2. Risk of Bias within Included Studies

The studies generally maintained a ‘Probably Low’ bias quality rating with respect to the exposure characterization, outcomes, outcome measures reported, statistical method and evaluation of confounding variables. Approximately 83% of the studies either employed a within-subject design or did not report if the participants or researchers were blind to the study group allocation (questions 1–3 in the OHAT tool). However, we note that a concealment of the study group during the study (question 3) is seldom feasible in light exposure studies, and that the lack of allocation concealment is unlikely to appreciably bias the results in studies within this field. Furthermore, studies using a within-participants design did blind the participants or use counterbalancing to minimize order effects. Approximately 17% of the studies excluded participants or outcome data from the experiment (question 4) due to insufficient data, poorly recorded data or algorithm removal (see Table 5). These actions were deemed unlikely to have increased the exclusion bias; thus, the overall risk of systematic bias was considered acceptable in all studies assessed, and no studies were excluded from the review based on the risk of bias criteria.

### 3.3. Study Characteristics

The characteristics of the twelve included studies, reporting outcomes for a total of 337 participants, are outlined in Table 6. The studies focused on individuals of good health and typically employed a small sample size (*M* = 28.08 participants, range = 4 to 112). The participants were aged between 18 and 65, with most studies reporting a mean age between 20 and 30. Four studies (33.33%) examined males only, two studies (16.67%) examined females only, and the remaining six studies examined approximately equal numbers of each sex. Cortisol was measured from the participants saliva in ten studies (83.33%), while two studies (16.67%) measured cortisol in blood, and melatonin was measured in five studies.

### 3.4. Light Exposure Characteristics

Light exposures were delivered via a variety of methods, including lamps, overhead room lighting and head-mounted devices, as noted and characterized in Table 7. The most common light conditions assessed in the included studies were red or red-enriched light, white (bright or dim defined by parameters outlined in each individual study) and blue or blue-enriched light, with one study employing green light. The perceived colour of light relates to its wavelength composition (i.e., spectral power distribution). All studies reported the peak wavelength (nm) of coloured experimental lighting conditions, and some provided additional information (such as spectrum graphs, half maximum bandwidth or CCT). The peak wavelengths for blue light ranged from 450 to 480 nm, red light ranged from 620 to 660 nm, and the peak wavelength of the green light condition tested was 520 nm. Few studies provided spectrum information for dim and bright light conditions.

Most studies compared one or more bright experimental lighting conditions with a dim broadband light baseline. All the studies reported the illuminance (lux), and eight studies (66.67%) also reported the irradiance (W/m^2^). The illuminance ranged from <2 lux in dim light conditions to 4300 lux in bright light conditions. In most studies, the experimental lighting conditions tested had an illuminance in the range of 200–550 lux, approximately equivalent to the brightness of typical indoor lighting. The experimental lighting exposure lengths ranged from 30 min to 3.5 h. Although most studies involved a single light exposure, some studies repeated the exposures throughout the day or across multiple days. The samples for cortisol measurement were typically collected at baseline (immediately prior to the light intervention) and then at multiple intervals within the 2 h following the beginning of the light exposure.

### 3.5. Study Results

#### 3.5.1. Comparison of Experimental Lighting Conditions with Pre-Exposure Baseline

**Figure 4:**
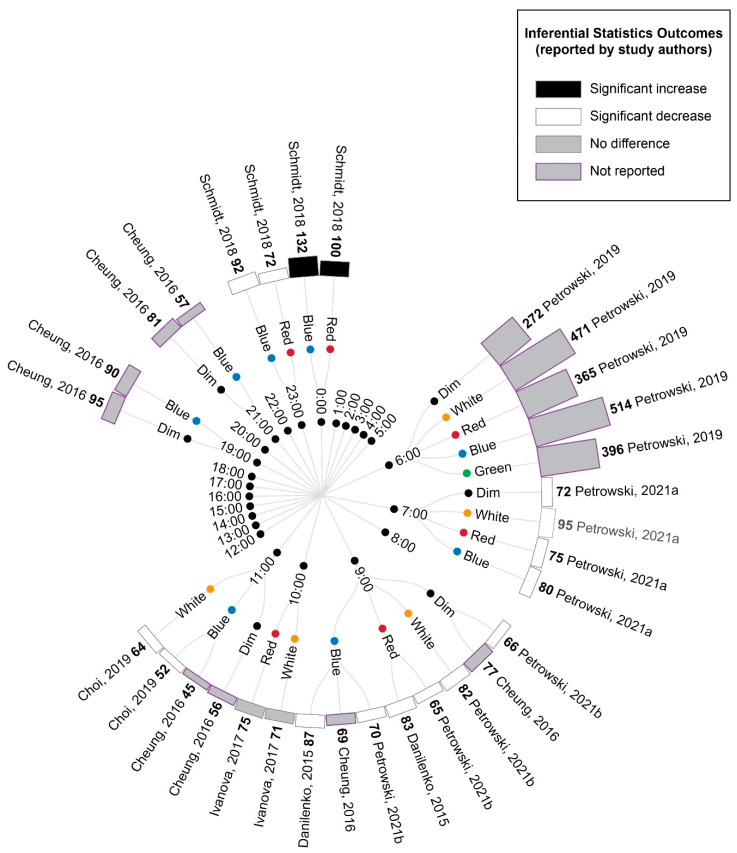
Radial tree displaying the percentage change in cortisol during or following experimental light interventions relative to a pre-light baseline. Inner nodes indicate the time of experimental cortisol measurement. Middle nodes indicate the type of light exposure (dim, white, red, blue or green light). The outer ring of nodes provides a bar graph indicating the percentage change in cortisol during or following the light exposure relative to a pre-light baseline. The percentage change is also displayed numerically in bold next to the study author’s name. Statistical significance as reported by the study authors is indicated by the bar shading and outline. Refer to Table 7 for author citations.

The extracted cortisol data were first converted to percentage difference measures by comparing the experimental light exposures with the pre-exposure baseline (Figure 4). These data demonstrated a pattern of increasing cortisol values relative to the baseline for samples collected between midnight and early in the morning, whereas samples collected from the early morning to evening showed decreasing cortisol values relative to the baseline, regardless of the experimental light condition being tested. This pattern is consistent with the expected circadian variations in cortisol levels across a day (see Figure 2 in Introduction).

#### 3.5.2. Comparison of Time-Matched Experimental Lighting Conditions

**Figure 5:**
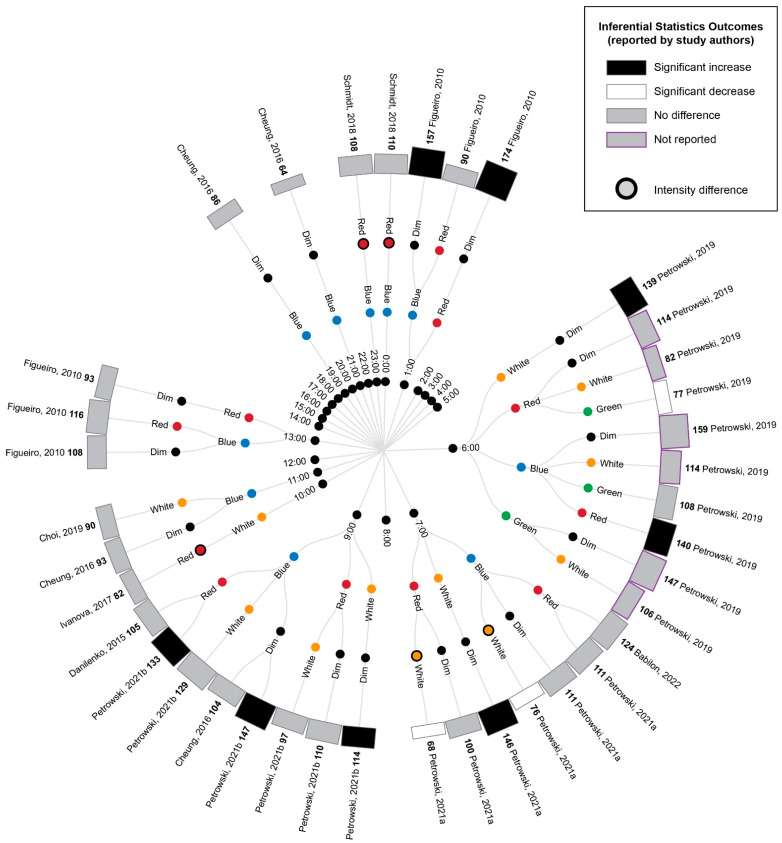
Radial tree displaying the percentage difference in cortisol measurements between time-matched experimental lighting conditions. The inner (first) nodes indicate the time of cortisol measurement (with minutes rounded to the nearest hour). Where multiple cortisol measurements were taken within a short timeframe, the data are summarized in 2 h. blocks. The second and third rings of nodes indicate the two experimental lighting conditions being compared (dim, white, red, blue or green light). In addition to the dim light controls, where coloured lights were substantially brighter or dimmer than their comparator counterparts, this is indicated with an additional black annulus around the coloured dot. The outer (fourth) ring of nodes provides a bar graph representing the percentage change in cortisol between the two experimental light exposures. The percentage change is also displayed numerically in bold next to the study author’s name. Statistical significance as reported by the study authors is indicated by the bar shading and outline. One study [45] only presented descriptive statistics for the average of cortisol measurements collected at 09:00, 13:00 and 17:00 (daytime) and 21:00, 01:00 and 05:00 (night-time). In this study, the night-time measures are expected to be affected by participant sleep deprivation. Two studies [40,74] reported insufficient descriptive statistics to enable calculation of percentage change in cortisol levels between conditions. Thus, their results were unable to be represented in this figure and are described in the manuscript text only. Refer to Table 7 for author citations.

A comparison of the cortisol measurements between time-matched experimental lighting conditions allows the effects of the light exposure characteristics, including spectral characteristics, to be assessed. Thus, the extracted cortisol data were next converted to percentage difference measures by comparing the time-matched experimental lighting conditions (Figure 5).

As illustrated in Figure 5, bright light of any kind (broadband white light, blue light or green light) generally elicited an increase in cortisol concentration relative to time-matched dim light exposure when measurements occurred between 21:00 p.m. and 9:00 a.m. and a decrease in cortisol concentration when measurements occurred between approximately 10:00 a.m. and 21:00 p.m. These differences were only statistically significant for the measurements conducted between 21:00 p.m. and 9:00 a.m., when four studies reported a significant increase in cortisol following blue, red and white light exposures relative to dim light exposures [9,41,42,45]. The single study examining green light did not statistically compare green and dim light conditions; however, the descriptive statistics provided demonstrated a difference of 147% between the conditions. This pattern of findings is consistent with the expectation that light exposure during the late evening or early morning can induce a phase advance in the circadian rhythm of cortisol production (which typically displays a nadir around midnight and a morning peak, as illustrated in Figure 2).

Due to the enhanced blue light sensitivity of ipRGCs, it has been hypothesized that light spectrums with strong short-wavelength contributions (e.g., blue light) may be more effective in inducing phase shifts in circadian rhythms, including cortisol rhythms, than light spectrums with strong long-wavelength contributions (e.g., red light). Consistent with this expectation, blue light exposures resulted in significantly greater cortisol levels relative to red light exposures at 6:00 and 9:00, green light resulted in significantly greater cortisol relative to red light at 6:00 and white light resulted in significantly greater cortisol relative to red light at 7:00. Contrary to the short-wavelength hypothesis, one study also found significantly lower cortisol following blue relative to white light exposure at 7:00; however, in this study, the white light condition was of a much higher illuminance (1240 lux) relative to the blue light condition (201 lux) [9].

Two studies provided insufficient descriptive statistics for their findings to be summarized in Figure 4 and Figure 5 [40,74]. Of these, Sahin et al. [74] found that white light and red light exposures during the day at 7:00, 11:00 and 15:00 did not significantly alter the cortisol measurements. Cai et al. [40] found that a 3.5 h exposure to blue-enriched lamps in the early evening for several days significantly increased the cortisol measurement profiles at 22:00–23:00 relative to lamps and room lights with relatively lower short-wavelength contributions. Thus, as with the comparisons to experimental dim light reported above, all the significant differences in cortisol between experimental bright light conditions occurred for measurements conducted between 21:00 and 9:00, which is consistent with the expectation that light exposures are most effective at inducing changes in cortisol levels in the late evening and early morning.

#### 3.5.3. Concurrent Melatonin Measurements

**Figure 6:**
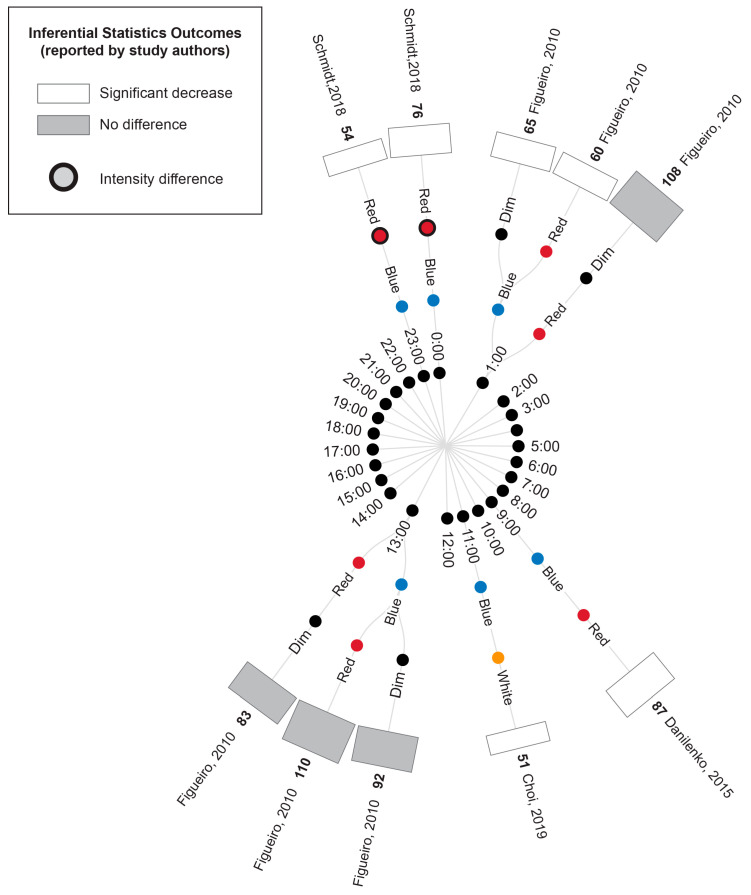
Radial tree displaying the percentage difference in melatonin measurements between time-matched experimental lighting conditions. The inner (first) nodes indicate the time of melatonin measurement (with minutes rounded to the nearest hour). Where multiple melatonin measurements were collected within a short timeframe, the data are summarized in 2 h. blocks. The second and third rings of nodes indicate the two experimental lighting conditions being compared (dim, white, red or blue light). In addition to the dim light controls indicated, where coloured lights were substantially brighter or dimmer than their comparator counterparts, this is indicated with an additional black annulus around the coloured dot. The outer (fourth) ring of nodes provides a bar graph indicating the percentage difference in melatonin between the two experimental light interventions (with the exact value indicated in bold above the bar) and the study author. Statistical significance as reported by the study authors is indicated by the bar shading and outline. One study [45] only presented descriptive statistics for the average of melatonin measurements collected at 09:00, 13:00 and 17:00 (daytime) and 21:00, 01:00 and 05:00 (night-time). In this study, the night-time measures were expected to be affected by participant sleep deprivation. One study [40] reported insufficient descriptive statistics to enable calculation of percentage change in melatonin levels between conditions. Thus, this study’s results were unable to be represented in the figure and are described in the manuscript text only. Refer to Table 7 for author citations.

Figure 6 illustrates the melatonin measures that were reported in conjunction with the cortisol results described above, where available. Two studies conducted morning light exposures (7:30 and 10:00). In these studies, blue/blue-enriched light produced a significantly greater suppression of melatonin compared to the time-matched warm white light [43] and red conditions [44]. As outlined above, neither study reported differences in cortisol concentration following exposure to these blue/blue-enriched lights relative to their red and white comparators.

Two studies conducted evening light exposures (19:00 and 22:00); in one study, blue light significantly suppressed melatonin concentration compared to a dimmer time-matched red light exposure, whereas there was no significant difference in cortisol levels between the two conditions [46]. The second study illustrated that melatonin was suppressed following exposure to blue-enriched lamps compared to lamps and room lighting with a lower short-wavelength contribution (with cortisol displaying an essentially opposite profile in the same study) [40].

One study examined light exposures distributed throughout the day [45]; this study observed an increase in melatonin overnight across all conditions (20:00, 00:00 and 4:00), with the melatonin concentration being significantly lower under the blue light condition compared to the time-matched dim and red-light conditions. However, the concurrent cortisol measures were greater for blue and red lights (relative to a dim light comparison), with no significant difference between the two coloured light conditions. Following daytime exposures (8:00, 12:00 and 16:00), melatonin stayed relatively low, showing a non-significant difference between the conditions, similar to cortisol measures.

## 4. Discussion

An optimization of the use of light in clinical interventions and in the design of constructed environments requires better understanding of how the HPA axis is influenced by different lighting parameters, including wavelength. It is known that the ipRGC-to-SCN (Retino-hypothalamic) pathway that entrains the central circadian system to light is particularly sensitive to intense short (blue) wavelengths [75]. However, whether this sensitivity translates into greater changes in downstream cortisol secretion following daytime exposures to blue lights (versus broadband white or red lights) remains unclear. Hence, the aim of the current systematic literature review was to evaluate research articles comparing the effect of daytime exposure to light of different wavelengths on HPA axis hormone secretion in the human body.

Following PRISMA protocols, 12 studies out of the 3418 references screened met the inclusion criteria for the review and were of acceptable quality, as determined using the OHAT risk of bias assessment tool. The synthesis of the eleven articles demonstrated that exposure to bright lights of any wavelength composition during the late evening to early morning typically increased cortisol secretion relative to dim or dark light comparison conditions at the same times and that light wavelength does influence the magnitude of the cortisol effect during this sensitive period. Brief light exposures did not significantly affect the cortisol measurements during the late morning to early evening, regardless of their wavelength composition.

### 4.1. Light Intensity and Wavelength Comparisons

Cortisol levels typically peak in the early morning and slowly decline throughout the day until approximately midnight. Consistent with this circadian rhythm of cortisol secretion, all the categories of bright light (white, blue, red, and green) induced increased cortisol secretion relative to the time-matched dark or dim light comparisons when the exposure occurred in the late evening or early morning when cortisol levels were increasing. The calculated percentage increase in cortisol elicited by bright light ranged from 114 to 174%, with values around 140–150% being the most typical. On the other hand, as expected, bright light exposure during the late morning to early evening, when cortisol levels are typically decreasing, did not significantly affect cortisol levels relative to the time-matched dim/dark light comparisons, though the studies measuring the effects of bright light at these time-points often reported a non-significant decrease in cortisol. This pattern of results is consistent with the expectation that bright light exposure can induce a phase shift in cortisol peak secretion in the early morning and that such light exposure near habitual sleep/wake times is most effective in inducing such a shift. Thus, the circadian system is more susceptible to light exposure or changes at these times in anticipation of the upcoming day and through reactions such as the CAR that occur in natural environments. It is possible that higher light intensities than those used within the studies in this review (where the illuminance of bright light conditions was typically between 200 and 550 lux) may have a stronger influence on the HPA axis function in the middle of the day [76].

In the late evening and early morning, the lights with strong shorter-wavelength components (e.g., blue or green light or very bright broad-wavelength white light [9]), typically induced greater increases in cortisol levels relative to lights with stronger long-wavelength (i.e., red light) components [9,40,41,42,45], though this difference was not always statistically significant [9,45]. Blue light is expected to stimulate ipRGCs that are hypersensitive to light wavelengths with a peak of 470–480 nm [75], and the present review suggests that this optimal stimulation of ipRGCs translates to greater effects on HPA axis pathway cortisol release relative to other types of light for the types of light exposures measured (typically continuous, short duration and moderate intensity).

The four studies that reported an accompanying significant change in melatonin suggest that, like cortisol, this hormone can be altered by short light exposures in the evening and morning. Consistent with the conclusions of previous systematic reviews [28,39], blue light elicited a relatively greater suppression of melatonin than red and white light [43,44,45,46]. This is an essentially opposite response to cortisol in the broader set of included studies, highlighting the role of the ipRGC-to-SCN pathway in regulating both hormones [77]. Interestingly, four of the five studies measuring both hormones reported wavelength-based differences in the melatonin measures but not in the cortisol measures, suggesting differences in measurement sensitivity, wavelength sensitivity or circadian timing sensitivity for the two hormones.

### 4.2. Limitations and Potential Bias in the Extracted Studies

There are several limitations that could affect the interpretation of the results of this review. Importantly, most studies did not justify their sample sizes and thus may have been underpowered. The sample sizes typically included under 50 participants and reported non-significant effects. Moreover, there could be a gender bias as 264 out of the 337 participants were male. Prior research suggests that there are sex differences in the circadian regulation of hormonal processes [78,79,80], including light-induced cortisol shifts [40]. The participants in the included studies were typically young adults (with most studies reporting a mean age of 20–30 years). Thus, further work is also needed to determine how the effects of light wavelength on cortisol effects vary across the lifespan, especially in children, where short-wavelength light is reported to minimize myopic shifts [81,82]. With regards to the delivery of the light interventions, the characterization of the light profiles was often incomplete. Most studies described the illuminance and peak wavelength of the experimental light condition/s but did not provide spectral power distribution graphs. Moreover, many studies provided no spectrum information for the dim light conditions.

### 4.3. Implications and Future Directions for Research

The present review suggests that the circadian regulation of cortisol, like melatonin, is sensitive to the wavelength composition of environmental lighting (with short wavelengths having a greater effect on cortisol levels, presumably because of the spectral tuning of ipRGCs). As such, the wavelength composition of the light source should be a key consideration when optimizing light treatments, and spectrum characteristics should be clearly reported in studies of interventions using light. Notably, blue-enriched light exposure in the early morning was reported by some studies to elicit an increase in cortisol levels of approximately 130–140% relative to longer-wavelength red light [9,41], an effect of similar magnitude to those previously associated with mental health disorders such as psychotic major depression [83]. Thus, these findings also highlight the potential for exposure to blue-enriched light during sensitive periods of the day to impact cortisol-associated disorders [84,85,86,87], and the associated need for light wavelength in constructed environments (such as bedrooms, workplaces and classrooms) to be carefully considered to optimize health and wellbeing. The spectrum and intensity of environmental light should also be considered in the design of studies exploring the HPA system. Systematic consideration of such factors would allow for greater exploration of the physiological processes underlying neuroendocrine-linked sleep, mood and cognitive conditions and disorders such as childhood myopia.

Further work is now needed to characterize the effects of light of different wavelengths on the circadian rhythm of cortisol more accurately. As summarized in Table 7 of the results, there was significant variation in the timeline of light delivery and cortisol measurement across the studies in this review. Most of the studies collected baseline (pre-exposure) cortisol measurements, followed by cortisol measurements at regular intervals in the two hours from light onset. Future research measuring the full circadian cycle of cortisol before and after light intervention could more precisely identify changes in the phase and amplitude of cortisol rhythms. More research is also needed to determine if cortisol changes induced by short light exposures are persistent and to determine the effect of different lighting protocols in terms of exposure duration and consecutive days of exposure, such as those reported by Cai et al. [40].

## 5. Conclusions

This systematic review has investigated the effects of exposure to light of different wavelengths on the cortisol output of the HPA axis in healthy individuals. The synthesis of available studies provided evidence that short exposures to white, blue, red and green light of similar intensity tended to increase cortisol secretion relative to a dim light control in the late evening to early morning but not at other timepoints. Blue light produced the largest increases compared to other types of bright light, suggesting that brief bright light exposures occurring near habitual sleep and wake times can induce a phase shift in diurnal physiological cortisol rhythms, consistent with the broader circadian literature. Furthermore, our review indicates that blue light, compared to other colours, is the most effective in inducing cortisol shifts when presented continuously for a short duration. The review also found evidence for the suppression of melatonin in blue light that was not always accompanied by significant changes in cortisol levels, highlighting a potential difference in the sensitivity of regulatory systems for these two hormones to light parameters. Future light intervention studies must consider wavelength, as well as other parameters (particularly intensity and circadian timing), in their design. Further research is needed to determine what length of light exposure is required to induce a change in the HPA axis function associated with cortisol and melatonin and how repeated light exposures (such as daily treatment) affect these hormonal parameters.

## Figures and Tables

**Table 1:** Database search strategy and example results from PubMed.

Search Terms	Search Results
“Light intensity” OR “light exposure” OR “artificial light” OR wavelength OR “violet light” OR “blue light” OR “green light” OR “yellow light” OR “red light” OR “light spectrum”.	155,092
HPA OR “HPA axis” OR “HPA activity” OR “HPA function” OR “hypothalamic pituitary adrenal” OR CRH OR “corticotropin releasing hormone” OR CRF OR “corticotropin releasing factor” OR ACTH OR “adrenocorticotropic hormone” OR CORT OR corticosterone OR cortisol OR glucocorticoid.	430,111
1 AND 2	597

Note: Search results are indicative of the number of studies available on 19 June 2023.

**Table 2:** Pre-specified hierarchy of exclusion criteria.

Pre-Specified Hierarchy of Exclusion Criteria	
1.	Full text not available (e.g., conference abstract).
2.	Study not published in English.
3.	Study population did not meet the inclusion criteria of healthy adult human, postnatal, without any known pre-existing disease (e.g., cancer, sleep deprivation).
4.	Study design was not observational (longitudinal, cross-sectional, book, qualitative, etc.).
5.	Insufficient light stimulus or HPA index detail.
6.	Does not include comparison of two or more clearly defined lighting conditions (e.g., light wavelength information not provided).
7.	Light exposure occurred only outside of the usual daylight photoperiod, as defined by the study authors (or 5:00–24:00, 24 h time, if not defined [56]).
8.	Does not include comparison of biochemical measures of HPA function across lighting conditions (i.e., corticosterone, cortisol, corticotropin releasing hormone and glucocorticoid).

Note: One study that utilized a stress manipulation to aid in measurement of HPA function was included in the review [57].

**Table 3:** OHAT questions used to assess risk of bias.

OHAT Risk of Bias Questions	
1.	Was administration dose or exposure level adequately randomized?
2.	Was allocation to study adequately concealed?
3.	Were the research personnel and human subjects blinded to the study group during the study?
4.	Were outcome data complete without attrition or exclusion from analysis?
5.	Can we be confident in the exposure characterization?
6.	Can we be confident in the outcome assessment?
7.	Were all measured outcomes reported?
8.	Were statistical methods appropriate?
9.	Did researchers adhere to the study protocol?
10.	Did the study design or analysis account for important confounding and modifying variables (unintended co-exposures) in experimental studies?

Note: All questions in the table originate from the “OHAT risk of bias rating tool for human and animal studies”, 2015.

**Table 4:** Details extracted from each of the studies included in the review.

Extracted Study Details	
1.	Study Information (author, year of publication)
2.	Study Aims
3.	Study Design
4.	Cortisol Collection Method
5.	Number of Participants
6.	Participants Age
7.	Participants Sex
8.	Light Conditions
9.	Light Correlated Colour Temperature (CCT)
10.	Light Irradiance
11.	Illuminance (lux)
12.	Spectral Power Distribution Characteristics (peak and bandwidth)
13.	Time of Light Exposure (24 h)
14.	Duration of Light Exposure
15.	Time of Sample Collection for Cortisol Measurement
16.	Cortisol Yield per Condition
17.	Time of Sample Collection for Melatonin Measurement (where applicable)
18.	Melatonin Yield per Condition (where applicable)

**Table 5:** OHAT risk of bias assessment table.

Author	Question									
	1	2	3	4	5	6	7	8	9	10
Babilon et al. [10]	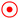	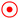	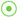	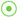	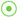	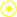	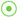	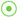	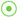	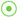
Cai et al. [40]	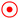	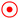	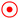	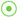	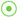	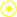	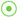	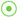	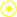	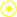
Cheung et al. [73]	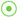	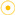	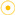	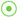	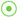	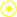	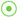	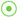	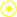	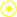
Choi et al. [43]	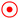	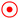	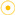	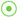	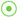	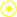	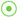	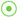	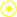	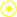
Danilenko et al. [44]	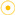	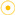	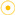	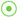	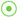	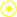	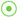	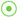	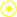	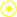
Figueiro et al. [45]	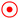	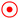	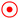	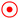	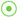	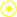	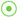	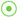	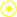	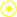
Ivanova et al. [57]	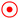	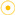	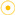	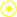	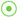	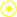	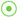	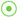	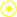	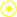
Petrowski et al. [41]	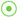	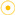	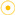	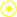	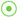	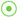	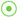	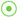	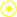	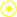
Petrowski at al. [9]	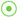	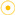	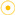	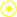	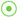	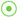	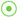	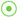	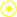	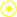
Petrowski et al. [42]	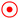	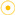	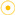	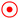	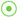	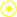	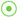	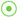	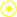	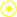
Sahin et al. [74]	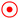	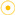	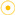	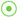	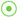	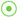	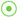	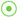	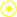	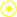
Schmidt et al. [46]	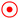	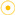	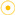	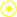	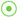	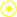	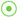	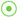	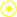	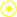

Note. 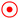 = Definitely high. 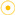 = Probably high. 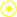 = Probably low. 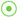 = Definitely low.

**Table 6:** Characteristics of included studies.

Author	Study Aim	Study Design	Participants			Cortisol Collection Method	Melatonin Collection Method
			*N*	Sex	Age: *M* (*SD*), range		
Babilon et al. [10]	To investigate the spectral dependency of morning cortisol secretion in humans using a consistent and practical method.	Within Participant	4	4 M	25.25 ± 3.59	Saliva	-
Cai et al. [40]	To investigate the effect of lamps with similar power and Correlated Colour Temperatures (CCTs) but distinct spectra on melatonin and cortisol secretion.	Within Participant	17	9 M, 8 F	21–30	Saliva	Saliva
Cheung et al. [73]	To determine the acute effects of morning versus evening, blue-enriched light exposure compared to dim light on hunger, metabolic function and physiological arousal.	Within Participant	19	8 M, 11 F	Morning group = 26.0 ± 4.4, Evening group = 29.9 ± 6.1, 20–39	Blood	-
Choi et al. [43]	To investigate physiological (melatonin, cortisol) and subjective responses to morning light exposure of lights with different CCTs.	Within Participant	15	8 M, 7 F	23.53 ± 3.37	Saliva	Saliva
Danilenko et al. [44]	To investigate the role of melanopsin-based photoreception in the effects of light on reproductive hormones in females.	Between Participants	16	16 F	28.0 ± 7.2, 20–44	Blood	Saliva
Figueiro et al. [45]	To investigate the impact of narrowband long-wavelength (red) and short-wavelength (blue) light exposures on the endocrine and autonomic systems (as measured by cortisol, alpha amylase and melatonin responses).	Within Participant	12	8 M, 4 F	19–53	Saliva	Saliva
Ivanova et al. [57]	To investigate the impact of light on metabolism.	Within Participants	10	10 F	44.9 ± 12.3, 22–59	Saliva	-
Petrowski et al. [41]	To investigate the influence of the spectral composition of light (brightness and wavelength) on the cortisol awakening response.	Within Participant	53 (30 in Study 1, 23 in Study 2)	53 M	Study 1 = 24.62 ± 3.32, Study 2 = 22.83 ± 3.33	Saliva	-
Petrowski at al. [9]	To investigate the effect of light intensity and spectral composition on the cortisol response after the Maastricht Acute Stress Test (MAST)	Between Participants	112	112 M	24.83 ± 4.10	Saliva	-
Petrowski et al. [42]	To compare the effects of short-wavelength blue light and bright light on cortisol secretion.	Within Participants	49 (23 in Study 1, 26 in Study 2)	49 M	Study 1 = 29.17 ± 7.57, Study 2 = 26.65 ± 7.00	Saliva	-
Sahin et al. [74]	To investigate the effects of daytime light exposure on performance, biomarkers and alertness.	Within Participant	13	6 M, 7 F	23 ± 5.5	Saliva	-
Schmidt et al. [46]	To investigate whether a blue-enriched light therapy device affects melatonin secretion, alertness, physiological stress and discomfort measures in the evening hours.	Within Participant	17	7 M, 8 F	22.8 ± 1.8	Saliva	Saliva

Note. M, F = Number of males and females. Partially complete cells are indicative of missing data.

**Table 7:** Light exposure characteristics.

Author	Condition	Intensity		Spectral Power Distribution				Light Exposure		Cortisol Measurement	Melatonin Measurement
		Lux	W/m^2^	Bandwidth	Peak (nm)	Half Max (nm)	CCT (K)	Start Time	Duration		
Babilon et al. [10]	Blue light	24 ± 3	30.29	Narrow	476	13	-	6:00	2 h	0, 20, 40, 60, 80 m	Not applicable
	Red light	22 ± 2	24.24	Narrow	649	14	-	6:00	2 h	0, 20, 40, 60, 80 m	Not applicable
Cai at al. [40]	Blue-enriched lamp 1	250 ± 50, 500 ± 50	-	Broad	453		5150	19:00	3.5 h	0, 3, 4, 12 h	0, 3, 4, 12 h
	Blue-enriched lamp 2	500 ± 50	-	Broad	467		5000	19:00	3.5 h	0, 3, 4, 12 h	0, 3, 4, 12 h
	Blue-enriched lamp 3	250 ± 50, 500 ± 50	-	Broad	453, 467		5050	19:00	3.5 h	0, 3, 4, 12 h	0, 3, 4, 12 h
	Dorm Lamp		-	Broad	450		4000	19:00	3.5 h	0, 3, 4, 12 h	0, 3, 4, 12 h
Cheung et al. [73]	Dim light	<20	-		-	-		From waking	16 h	0, 0.5, 1, 1.5, 2, 2.5, 3, 3.5, 4 h	Not-applicable
	Blue-enriched light	260 (Overhead), 370 (Blue)	-	Narrow blue and broad overhead lighting	468 ± 8 (narrowband blue)	-		07:50 or 17:45	3 h	0, 0.5, 1, 1.5, 2, 2.5, 3, 3.5, 4 h	Not applicable
Choi et al. [43]	Dim light	<10	-	Dynamic (film)	-	-		9:00 (baseline)	1 h	-	-
	Warm white light	516.14	1.641	Broad	625	-	3590	10:00	1 h	0, 1 h	0, 1 h
	Blue-enriched white light	518.38	1.793	Broad	460	-	6575	10:00	1 h	0, 1 h	0, 1 h
Danilenko et al. [44]	Dim light (sunglasses during walk to study location)	<10	-	-	-	-	-	-	10–15 m	-	-
	Red light	1100	7	Narrow	651	-	-	7:30	45 m	0, 22, 44 m	0, 22, 44 m
	Blue-enriched white light	1300	7	Broad	469	-	-	7:30	45 m	0, 22, 44 m	0, 22, 44 m
Figueiro et al. [45]	Dim light	<3	-	-	-	-	-	8:00, 12:00, 16:00, 20:00, 00:00, 04:00, 08:00	1 h	0, 1 h	0, 1 h
	Red light	40	0.19	Narrow	625	25	-	8:00, 12:00, 16:00, 20:00, 00:00, 04:00, 08:00	1 h	0, 1 h	0, 1 h
	Blue light	40	0.4	Narrow	470	25	-	8:00, 12:00, 16:00, 20:00, 00:00, 04:00, 08:00	1 h	0, 1 h	0, 1 h
Ivanova et al. [57]	Dim light	<100	-	-	-	-	-	8:30 (pre-experiment baseline)	15 m	-	Not applicable
	Red light	250	-	Narrow	620	-	-	9:00	30 m	0, 30, 45 m	Not applicable
	Bright white light	4300	-	Broad	550 and 620	-	-	9:00	30 m	0, 30, 45 m	Not applicable
Petrowski et al. [41]	Dim light	<2	-	-	-	-	-	5:05	1 h	0, 15, 30, 45, 60, 75, 90, 105 m	Not applicable
	Bright white light	414	1.566	RGB	-	-	-	5:05	1 h	0, 15, 30, 45, 60, 75, 90, 105 m	Not applicable
	Red light	235	1.341	Narrow	635	-	-	5:05	1 h	0, 15, 30, 45, 60, 75, 90, 105 m	Not applicable
	Blue light	201	1.76	Narrow	475	-	-	5:05	1 h	0, 15, 30, 45, 60, 75, 90, 105 m	Not applicable
	Green light	806	1.598	Narrow	520	-	-	5:05	1 h	0, 15, 30, 45, 60, 75, 90, 105 m	Not applicable
Petrowski at al. [9]	Dim light	<2	1.432	RGB	-	-	-	6:00	1 h	−15, −10, 0, 15, 30, 45, 60, 75, 90 m	Not applicable
	Bright white light	1240	1.566	RGB	-	-	-	6:00	1 h	−15, −10, 0, 15, 30, 45, 60, 75, 90 m	Not applicable
	Red light	235	1.341	Narrow	635	-	-	6:00	1 h	−15, −10, 0, 15, 30, 45, 60, 75, 90 m	Not applicable
	Blue light	201	1.76	Narrow	470–480	-	-	6:00	1 h	−15, −10, 0, 15, 30, 45, 60, 75, 90 m	Not applicable
Petrowski et al. [42]	Dim light	<2	-	-	-	-	-	6:30 (baseline)	1 h	-	Not applicable
	Bright white light	414	-	-	-	-	-	7:30	1 h	0, 15, 30, 45, 60, 75, 90, 105 m	Not applicable
	Red light	235	-	Narrow	635	-	-	7:30	1 h	0, 15, 30, 45, 60, 75, 90, 105 m	Not applicable
	Blue light	201	-	Narrow	470–480	-	-	7:30	1 h	0, 15, 30, 45, 60, 75, 90, 105 m	Not applicable
Sahin et al. [74]	Dim light	<5		Fluorescent	-	-	3500	6:00 (baseline) then 7:00, 11:00, 15:00	1 h (baseline), 2 h (experimental)	−10, 50, 110 m	Not applicable
	Red light glasses	213	1.1	-	631	16	-	7:00, 11:00, 15:00	2 h	−10, 50, 110 m	Not applicable
	White light glasses	361	1.12	-	-	-	2568	7:00, 11:00, 15:00	2 h	−10, 50, 110 m	Not applicable
Schmidt et al. [46]	Dim light	<5	-	-	-	-	-	18:00	5 h	-	-
	Head-mounted red light	150	2.75	Narrowband	660	-	-	22:00	2 h	−4, −3, −2, −1, 0, 0.5, 1, 1.5, 2, 2.5, 3, 3.5 h	−4, −3, −2, −1, 0, 0.5, 1, 1.5, 2, 2.5, 3, 3.5 h
	Head-mounted, blue-enriched light	1500	4.66	Broadband	460	-	-	22:00	2 h	−4, −3, −2, −1, 0, 0.5, 1, 1.5, 2, 2.5, 3, 3.5 h	−4, −3, −2, −1, 0, 0.5, 1, 1.5, 2, 2.5, 3, 3.5 h

Note: Lux = illuminance in lux. W/m^2^ = irradiance in watts per square meter. Peak = spectral power distribution peak bandwidth. Half max = width of spectrum at half the maximum amplitude. CCT (K) = Correlated Colour Temperature in kelvin. Cort measurement = timing of cortisol measurement relative to the start of the light exposure (minus values indicate measurements prior to light exposure). Partially complete or empty cells are indicative of missing data.

## Supplementary Materials

The following supporting information can be downloaded at: https://www.mdpi.com/article/10.3390/life13101968/s1, File 1: PRISMA checklist, Table S1: Study Results.

## References

[1] Brabant G., Henley D.E., Kaye J.M., Lightman S.L. (2011). 2.6.1 Endocrinology, sleep and circadian rhythms. Oxford Textbook of Endocrinology and Diabetes.

[2] Androulakis I.P. (2021). Circadian rhythms and the HPA axis: A systems view. WIREs Mech. Dis..

[3] Ishida A., Mutoh T., Ueyama T., Bando H., Masubuchi S., Nakahara D., Tsujimoto G., Okamura H. (2005). Light activates the adrenal gland: Timing of gene expression and glucocorticoid release. Cell Metab..

[4] Buijs R.M., Wortel J., Van Heerikhuize J.J., Feenstra M.G., Ter Horst G.J., Romijn H.J., Kalsbeek A. (1999). Anatomical and functional demonstration of a multisynaptic suprachiasmatic nucleus adrenal (cortex) pathway. Eur. J. Neurosci..

[5] Lyall L.M., Wyse C.A., Graham N., Ferguson A., Lyall D.M., Cullen B., Celis Morales C.A., Biello S.M., Mackay D., Ward J. (2018). Association of disrupted circadian rhythmicity with mood disorders, subjective wellbeing, and cognitive function: A cross-sectional study of 91 105 participants from the UK Biobank. Lancet Psychiatry.

[6] Ayyar V.S., Sukumaran S. (2021). Circadian rhythms: Influence on physiology, pharmacology, and therapeutic interventions. J. Pharmacokinet. Pharmacodyn..

[7] Auld F., Maschauer E.L., Morrison I., Skene D.J., Riha R.L. (2017). Evidence for the efficacy of melatonin in the treatment of primary adult sleep disorders. Sleep Med. Rev..

[8] Webb A.R. (2006). Considerations for lighting in the built environment: Non-visual effects of light. Energy Build..

[9] Petrowski K., Buehrer S., Niedling M., Schmalbach B. (2021). The effects of light exposure on the cortisol stress response in human males. Stress.

[10] Babilon S., Myland P., Klabes J., Simon J., Khanh T.Q. (2022). Study protocol for measuring the impact of (quasi-)monochromatic light on post-awakening cortisol secretion under controlled laboratory conditions. PLoS ONE.

[11] Bellia L., Bisegna F., Spada G. (2011). Lighting in indoor environments: Visual and non-visual effects of light sources with different spectral power distributions. Build. Environ..

[12] Do A., Li V.W., Huang S., Michalak E.E., Tam E.M., Chakrabarty T., Yatham L.N., Lam R.W. (2022). Blue-Light Therapy for Seasonal and Non-Seasonal Depression: A Systematic Review and Meta-Analysis of Randomized Controlled Trials. Can. J. Psychiatry.

[13] Faulkner S.M., Bee P.E., Meyer N., Dijk D.J., Drake R.J. (2019). Light therapies to improve sleep in intrinsic circadian rhythm sleep disorders and neuro-psychiatric illness: A systematic review and meta-analysis. Sleep. Med. Rev..

[14] Calvo-Sanz J.A., Tapia-Ayuga C.E. (2020). Blue light emission spectra of popular mobile devices: The extent of user protection against melatonin suppression by built-in screen technology and light filtering software systems. Chronobiol. Int..

[15] Good P.A., Taylor R.H., Mortimer M.J. (1991). The use of tinted glasses in childhood migraine. Headache.

[16] Adams W.H., Digre K.B., Patel B.C., Anderson R.L., Warner J.E., Katz B.J. (2006). The evaluation of light sensitivity in benign essential blepharospasm. Am. J. Ophthalmol..

[17] Williams G.J., Kitchener G., Press L.J., Scheiman M.M., Steele G.T. (2004). The use of tinted lenses and colored overlays for the treatment of dyslexia and other related reading and learning disorders. Optometry.

[18] Berson D.M., Dunn F.A., Takao M. (2002). Phototransduction by retinal ganglion cells that set the circadian clock. Science.

[19] Hastings M.H., Maywood E.S., Brancaccio M. (2018). Generation of circadian rhythms in the suprachiasmatic nucleus. Nat. Rev. Neurosci..

[20] Pfeffer M., Korf H.W., Wicht H. (2018). Synchronizing effects of melatonin on diurnal and circadian rhythms. Gen. Comp. Endocrinol..

[21] Rao R., Androulakis I.P. (2019). The physiological significance of the circadian dynamics of the HPA axis: Interplay between circadian rhythms, allostasis and stress resilience. Horm. Behav..

[22] Pandi-Perumal S.R., Srinivasan V., Spence D.W., Cardinali D.P. (2007). Role of the Melatonin System in the Control of Sleep. CNS Drugs.

[23] Oster H., Challet E., Ott V., Arvat E., de Kloet E.R., Dijk D.J., Lightman S., Vgontzas A., Van Cauter E. (2017). The Functional and Clinical Significance of the 24-Hour Rhythm of Circulating Glucocorticoids. Endocr. Rev..

[24] Clow A., Hucklebridge F., Thorn L., Clow A., Thorn L. (2010). The Cortisol Awakening Response in Context. International Review of Neurobiology.

[25] Cuesta M., Cermakian N., Boivin D.B. (2015). Glucocorticoids entrain molecular clock components in human peripheral cells. Faseb. J..

[26] Montaruli A., Castelli L., Mulè A., Scurati R., Esposito F., Galasso L., Roveda E. (2021). Biological Rhythm and Chronotype: New Perspectives in Health. Biomolecules.

[27] Hickie I.B., Naismith S.L., Robillard R., Scott E.M., Hermens D.F. (2013). Manipulating the sleep-wake cycle and circadian rhythms to improve clinical management of major depression. BMC Med..

[28] Tähkämö L., Partonen T., Pesonen A.-K. (2019). Systematic review of light exposure impact on human circadian rhythm. Chronobiol. Int..

[29] Vondrašová D., Hájek I., Illnerová H. (1997). Exposure to long summer days affects the human melatonin and cortisol rhythms. Brain Res..

[30] Hofman M.A., Swaab D.F. (1993). Diurnal and Seasonal Rhythms of Neuronal Activity in the Suprachiasmatic Nucleus of Humans. J. Biol. Rhythm..

[31] Münch M., Bromundt V. (2012). Light and chronobiology: Implications for health and disease. Dialogues Clin. Neurosci..

[32] Zeitzer J.M., Dijk D.J., Kronauer R., Brown E., Czeisler C. (2000). Sensitivity of the human circadian pacemaker to nocturnal light: Melatonin phase resetting and suppression. J. Physiol..

[33] Phillips A.J.K., Vidafar P., Burns A.C., McGlashan E.M., Anderson C., Rajaratnam S.M.W., Lockley S.W., Cain S.W. (2019). High sensitivity and interindividual variability in the response of the human circadian system to evening light. Proc. Natl. Acad. Sci. USA.

[34] Sliney D.H. (2016). What is light? The visible spectrum and beyond. Eye.

[35] Brainard G.C., Hanifin J.P., Greeson J.M., Byrne B., Glickman G., Gerner E., Rollag M.D. (2001). Action spectrum for melatonin regulation in humans: Evidence for a novel circadian photoreceptor. J. Neurosci..

[36] Cajochen C., Jud C., Münch M., Kobialka S., Wirz-Justice A., Albrecht U. (2006). Evening exposure to blue light stimulates the expression of the clock gene PER2 in humans. Eur. J. Neurosci..

[37] Gooley J.J., Rajaratnam S.M., Brainard G.C., Kronauer R.E., Czeisler C.A., Lockley S.W. (2010). Spectral responses of the human circadian system depend on the irradiance and duration of exposure to light. Sci. Transl. Med..

[38] Rahman S.A., Brainard G.C., Czeisler C.A., Lockley S.W. (2021). Spectral sensitivity of circadian phase resetting, melatonin suppression and acute alerting effects of intermittent light exposure. Biochem. Pharmacol..

[39] Xiao H., Cai H., Li X. (2021). Non-visual effects of indoor light environment on humans: A review(✰). Physiol. Behav..

[40] Cai J., Hao W., Zeng S., Qu X., Guo Y., Tang S., An X., Luo A. (2021). Effects of Red Light on Circadian Rhythm: A Comparison Among Lamps With Similar Correlated Color Temperatures Yet Distinct Spectrums. IEEE Access.

[41] Petrowski K., Schmalbach B., Niedling M., Stalder T. (2019). The effects of post-awakening light exposure on the cortisol awakening response in healthy male individuals. Psychoneuroendocrinology.

[42] Petrowski K., Bührer S., Albus C., Schmalbach B. (2021). Increase in cortisol concentration due to standardized bright and blue light exposure on saliva cortisol in the morning following sleep laboratory. Stress.

[43] Choi K., Shin C., Kim T., Chung H.J., Suk H.-J. (2019). Awakening effects of blue-enriched morning light exposure on university students’ physiological and subjective responses. Sci. Rep..

[44] Danilenko K.V., Sergeeva O.Y. (2015). Immediate effect of blue-enhanced light on reproductive hormones in women. Neuro Endocrinol. Lett..

[45] Figueiro M.G., Rea M.S. (2010). The Effects of Red and Blue Lights on Circadian Variations in Cortisol, Alpha Amylase, and Melatonin. Int. J. Endocrinol..

[46] Schmidt C., Xhrouet M., Hamacher M., Delloye E., LeGoff C., Cavalier E., Collette F., Vandewalle G. (2018). Light exposure via a head-mounted device suppresses melatonin and improves vigilant attention without affecting cortisol and comfort. Psych. J..

[47] Johnson J.A., Subnis U., Carlson L.E., Garland S.N., Santos-Iglesias P., Piedalue K.-A.L., Deleemans J.M., Campbell T.S. (2020). Effects of a light therapy intervention on diurnal salivary cortisol in fatigued cancer survivors: A secondary analysis of a randomized controlled trial. J. Psychosom. Res..

[48] Ancoli-Israel S., Rissling M., Neikrug A., Trofimenko V., Natarajan L., Parker B.A., Lawton S., Desan P., Liu L. (2012). Light treatment prevents fatigue in women undergoing chemotherapy for breast cancer. Support. Care Cancer.

[49] Bower J.E., Ganz P.A., Dickerson S.S., Petersen L., Aziz N., Fahey J.L. (2005). Diurnal cortisol rhythm and fatigue in breast cancer survivors. Psychoneuroendocrinology.

[50] Agustini B., Bocharova M., Walker A.J., Berk M., Young A.H., Juruena M.F. (2019). Has the sun set for seasonal affective disorder and HPA axis studies? A systematic review and future prospects. J. Affect. Disord..

[51] Buckley T.M., Schatzberg A.F. (2005). On the interactions of the hypothalamic-pituitary-adrenal (HPA) axis and sleep: Normal HPA axis activity and circadian rhythm, exemplary sleep disorders. J. Clin. Endocrinol. Metab..

[52] Nandam L.S., Brazel M., Zhou M., Jhaveri D.J. (2019). Cortisol and Major Depressive Disorder-Translating Findings From Humans to Animal Models and Back. Front. Psychiatry.

[53] van Maanen A., Meijer A.M., van der Heijden K.B., Oort F.J. (2016). The effects of light therapy on sleep problems: A systematic review and meta-analysis. Sleep Med. Rev..

[54] Page M.J., McKenzie J.E., Bossuyt P.M., Boutron I., Hoffmann T.C., Mulrow C.D., Shamseer L., Tetzlaff J.M., Akl E.A., Brennan S.E. (2021). The PRISMA 2020 statement: An updated guideline for reporting systematic reviews. PLoS Med..

[55] Bramer W.M., Rethlefsen M.L., Kleijnen J., Franco O.H. (2017). Optimal database combinations for literature searches in systematic reviews: A prospective exploratory study. Syst. Rev..

[56] Scheuermaier K., Laffan A.M., Duffy J.F. (2010). Light Exposure Patterns in Healthy Older and Young Adults. J. Biol. Rhythm..

[57] Ivanova I.A., Danilenko K.V., Aftanas L.I. (2016). Investigation of an Immediate Effect of Bright Light on Oxygen Consumption, Heart Rate, Cortisol, and α-Amylase in Seasonal Affective Disorder Subjects and Healthy Controls. Neuropsychobiology.

[58] Mitchell M. Engauge Digitizer Software. http://markummitchell.github.io/engauge-digitizer.

[59] Houston D.C.R. Flourish. https://flourish.studio/.

[60] Petrowski K., Schmalbach B., Stalder T. (2019). The effects of light exposure on the cortisol awakening response in healthy individuals. Psychoneuroendocrinology.

[61] Traut J., Prius Mengua J., Meijer E.J., McKillop L.E., Alfonsa H., Hoerder-Suabedissen A., Song S.M., Molnár Z., Akerman C.J., Vyazovskiy V.V. (2022). Oral Abstract. J. Sleep Res..

[62] Partonen T., Meesters A.N., Maukonen M., Männistö S., Gordijn M.C., Meesters Y., Danilenko K.V., Ivanova I.A., Aftanas L.I., Gbyl K. (2016). Abstracts. Neuropsychobiology.

[63] Kashyap K.C., Nissen L., Smith S.S., Kyle G. (2012). Abstracts. J. Sleep Res..

[64] Petrowski K., Schmallbach B., Stalder T. (2019). The effects of light exposure on the cortisol awakening response. Psychoneuroendocrinology.

[65] Garefelt J., Akerstedt T., Westerlund H., Hanson L.M., Sverke M., Kecklund G. (2014). Abstracts. J. Sleep Res..

[66] Rahman S., Marcu S., Shapiro C., Brown T., Casper R. Attenuating Nocturnal Light Induced Disruption in Endocrine, Genetic and Behavioral Circadian Rhythm Phase Markers by Filtering Short Wavelengths. Proceedings of the 23rd Annual Meeting of the Associated-Professional-Sleep-Societies.

[67] Takemura Y., Kido K., Kawana H., Yamamoto T., Sanuki T., Mukai Y. (2021). Effects of Green Color Exposure on Stress, Anxiety, and Pain during Peripheral Intravenous Cannulation in Dental Patients Requiring Sedation. Int. J. Environ. Res. Public Health.

[68] Ba-Ali S., Brøndsted A.E., Andersen H.U., Sander B., Jennum P.J., Lund-Andersen H. (2019). Assessment of diurnal melatonin, cortisol, activity, and sleep−wake cycle in patients with and without diabetic retinopathy. Sleep Med..

[69] Gabel V., Maire M., Reichert C.F., Chellappa S.L., Schmidt C., Hommes V., Viola A.U., Cajochen C. (2013). Effects of Artificial Dawn and Morning Blue Light on Daytime Cognitive Performance, Well-being, Cortisol and Melatonin Levels. Chronobiol. Int..

[70] Komada Y., Aoki K., Gohshi S., Ichioka H., Shibata S. (2015). Effects of television luminance and wavelength at habitual bedtime on melatonin and cortisol secretion in humans. Sleep Biol. Rhythm..

[71] Schmid S.R., Höhn C., Bothe K., Plamberger C.P., Angerer M., Pletzer B., Hoedlmoser K. (2021). How Smart Is It to Go to Bed with the Phone? The Impact of Short-Wavelength Light and Affective States on Sleep and Circadian Rhythms. Clocks Sleep.

[72] Makateb A., Rashidinia A., Khosravifard K., Dabaghi P. (2023). Investigating the effects of a blue-blocking software on the daily rhythm of sleep, melatonin, cortisol, positive and negative emotions. Chronobiol. Int..

[73] Cheung I.N., Zee P.C., Shalman D., Malkani R.G., Kang J., Reid K.J. (2016). Morning and Evening Blue-Enriched Light Exposure Alters Metabolic Function in Normal Weight Adults. PLoS ONE.

[74] Sahin L., Wood B.M., Plitnick B., Figueiro M.G. (2014). Daytime light exposure: Effects on biomarkers, measures of alertness, and performance. Behav. Brain Res..

[75] Wahl S., Engelhardt M., Schaupp P., Lappe C., Ivanov I.V. (2019). The inner clock—Blue light sets the human rhythm. J. Biophotonics.

[76] Levitan R.D. (2005). What is the optimal implementation of bright light therapy for seasonal affective disorder (SAD)?. J. Psychiatry Neurosci..

[77] Milosavljevic N., Cehajic-Kapetanovic J., Procyk C.A., Lucas R.J. (2016). Chemogenetic Activation of Melanopsin Retinal Ganglion Cells Induces Signatures of Arousal and/or Anxiety in Mice. Curr. Biol..

[78] Duffy J.F., Cain S.W., Chang A.-M., Phillips A.J.K., Münch M.Y., Gronfier C., Wyatt J.K., Dijk D.-J., Wright K.P., Czeisler C.A. (2011). Sex difference in the near-24-hour intrinsic period of the human circadian timing system. Proc. Natl. Acad. Sci. USA.

[79] Horrocks P.M., Jones A.F., Ratcliffe W.A., Holder G., White A., Holder R., Ratcliffe J.G., London D.R. (1990). Patterns of ACTH and cortisol pulsatility over twenty-four hours in normal males and females. Clin. Endocrinol..

[80] Gunn P.J., Middleton B., Davies S.K., Revell V.L., Skene D.J. (2016). Sex differences in the circadian profiles of melatonin and cortisol in plasma and urine matrices under constant routine conditions. Chronobiol. Int..

[81] Torii H., Mori K., Okano T., Kondo S., Yang H.Y., Yotsukura E., Hanyuda A., Ogawa M., Negishi K., Kurihara T. (2022). Short-Term Exposure to Violet Light Emitted from Eyeglass Frames in Myopic Children: A Randomized Pilot Clinical Trial. J. Clin. Med..

[82] Thakur S., Dhakal R., Verkicharla P.K. (2021). Short-Term Exposure to Blue Light Shows an Inhibitory Effect on Axial Elongation in Human Eyes Independent of Defocus. Investig. Ophthalmol. Vis. Sci..

[83] Cherian K., Schatzberg A.F., Keller J. (2019). HPA axis in psychotic major depression and schizophrenia spectrum disorders: Cortisol, clinical symptomatology, and cognition. Schizophr. Res..

[84] Hu H., Kang C., Hou X., Zhang Q., Meng Q., Jiang J., Hao W. (2020). Blue Light Deprivation Produces Depression-Like Responses in Mongolian Gerbils. Front. Psychiatry.

[85] Keller J., Flores B., Gomez R.G., Solvason H.B., Kenna H., Williams G.H., Schatzberg A.F. (2006). Cortisol circadian rhythm alterations in psychotic major depression. Biol. Psychiatry.

[86] Germain A., Kupfer D.J. (2008). Circadian rhythm disturbances in depression. Hum. Psychopharmacol..

[87] Adam E.K., Vrshek-Schallhorn S., Kendall A.D., Mineka S., Zinbarg R.E., Craske M.G. (2014). Prospective associations between the cortisol awakening response and first onsets of anxiety disorders over a six-year follow-up--2013 Curt Richter Award Winner. Psychoneuroendocrinology.

