# Supplementary material for: The Influence of Light Wavelength on Human HPA Axis Rhythms: A Systematic Review
Source: Life (Basel). 2023 Sep 26;13(10):1968. doi: 10.3390/life13101968 (PMC10608196; doi:10.3390/life13101968)
Supplement: Supplementary file 1 [file life-13-01968-s001.zip › 230926 Updated Supplementary Files/Supplementary Table 1- Study Results.pdf]

Supplementary Table Providing Summary Statistics for Each Group. For each study included in the systematic review, this table presents the sample size of each group and the mean and standard deviation of cortisol measurements. If this information was not available, alternative descriptive statistics are provided where possible.

Author	Light Condition	N	Mean Cortisol Yield (SE or SD)
Babilon et al. 2022 [10]	Red Light	4	9.68 (SEM 1.21) nmol L^{-1}
	Blue Light		12.04 (SEM 1.54) nmol L^{-1}
Cai et al. 2021 [40]	Blue-enriched Lamp 1	17	Not Available
	Blue-enriched Lamp 2		
	Blue-enriched Lamp 3		
	Dorm Lamp		
Cheung et al. 2016 [73]	Blue Light	19	Morning: 2178 (SD 408) mg/dL<br>Evening: 972 (SD 438) mg/dL
	Dim Light		Morning: 2292 (SD 711) mg/dL<br>Evening: 1206 (SD 741) mg/dL
Choi et al. 2019 [43]	White Light	15	Percentage Decrease:<br>13.36% (SEM 20.88)
	Blue Light		Percentage Decrease:<br>43% (SEM 6.12)
Danilenko et al. 2015 [44]	White Light	10	Median (10th and 90th percentiles):<br>0 min: 638 (510–763) nmol/l<br>22 min: 581 (436–763) nmol/l<br>44 min: 501 (367–641) nmol/l
	Red Light	6	Median (10th and 90th percentiles):<br>0 min: 640 (479–842) nmol/l<br>22 min: 617 (395–707) nmol/l<br>44 min: 478 (372–631) nmol/l
Figueiro et al. 2010 [45]	Red Light	12	Mean normalized concentration:<br>Day: 0.29<br>Night: 0.31
	Blue Light		Mean normalized concentration:<br>Day: 0.34<br>Night: 0.28
	Dark		Mean normalized concentration:<br>Day: 0.32<br>Night: 0.18
Ivanova et al. 2017 [57]	White Light	10	0 min: 6.31 ng/mL<br>30 min: 5.30 ng/mL<br>55 min: 3.71 ng/mL
	Red Light		0 min: 7.27 ng/mL<br>30 min: 5.00 ng/mL<br>55 min: 5.96 ng/mL
Petrowski et al. 2021 [9]	Bright Light	27	11.40 (3.70) nmol/l
	Red Light	31	8.51 (3.33) nmol/l
	Blue Light	28	9.21 (3.62) nmol/l
	Dim Light	25	8.63 (5.28) nmol/l

Author	Light Condition	N	Mean Cortisol Yield (SE or SD)
Petrowski at al. 2021 [42]	Bright Light	49	9.72 (SD 5.97) nmol/l
	Red Light		9.79 (SD 4.82) nmol/l
	Blue Light		12.86 (SD 6.00) nmol/l
	Dim Light		8.85 (SD 4.70) nmol/l
Petrowski et al. 2019 [41]	Bright Light	30	11.41 (SD 5.07) nmol/l
	Dim Light		8.40 (SD 4.54) nmol/l
	Blue Light	23	13.02 (SD 5.97) nmol/l
	Green Light		12.15 (SD 5.96) nmol/l
	Red Light		9.43 (SD 5.53) nmol/l
Sahin et al. 2014 [74]	White Light Glasses	13	Not Available
	Red Light Glasses		
	Dim Light		
Schmidt et al. 2018 [46]	Red Light	17	Pre-light:<br>0.65 ug/L<br>0.55 ug/L<br>0.53 ug/L<br>0.35 ug/L<br>0.24 ug/L<br><br>Light:<br>0.21 ug/L<br>0.27 ug/L<br>0.30 ug/L<br>0.29 ug/L<br><br>Post-light:<br>0.31 ug/L<br>0.40 ug/L<br>0.42 ug/L
	Blue Light		Pre-light:<br>0.75 ug/L<br>0.43 ug/L<br>0.37 ug/L<br>0.34 ug/L<br>0.23 ug/L<br><br>Light:<br>0.23 ug/L<br>0.29 ug/L<br>0.36 ug/L<br>0.28 ug/L<br><br>Post-light:<br>0.29 ug/L<br>0.41 ug/L<br>0.54 ug/L

References

10. Babilon, S.; Myland, P.; Klabes, J.; Simon, J.; Khanh, T.Q. Study protocol for measuring the impact of (quasi-)monochromatic light on post-awakening cortisol secretion under controlled laboratory conditions. *PLOS ONE* **2022**, *17*, e0267659, doi:10.1371/journal.pone.0267659.

40. Cai, J.; Hao, W.; Zeng, S.; Qu, X.; Guo, Y.; Tang, S.; An, X.; Luo, A. Effects of Red Light on Circadian Rhythm: A Comparison Among Lamps With Similar Correlated Color Temperatures Yet Distinct Spectrums. *IEEE Access* **2021**, *9*, 59222-59230, doi:10.1109/ACCESS.2021.3073102.

73. Cheung, I.N.; Zee, P.C.; Shalman, D.; Malkani, R.G.; Kang, J.; Reid, K.J. Morning and Evening Blue-Enriched Light Exposure Alters Metabolic Function in Normal Weight Adults. *PLOS ONE* **2016**, *11*, e0155601, doi:10.1371/journal.pone.0155601.

43. Choi, K.; Shin, C.; Kim, T.; Chung, H.J.; Suk, H.-J. Awakening effects of blue-enriched morning light exposure on university students' physiological and subjective responses. *Scientific Reports* **2019**, *9*, 345, doi:10.1038/s41598-018-36791-5.

44. Danilenko, K.V.; Sergeeva, O.Y. Immediate effect of blue-enhanced light on reproductive hormones in women. *Neuro Endocrinol Lett* **2015**, *36*, 84-90.

45. Figueiro, M.G.; Rea, M.S. The Effects of Red and Blue Lights on Circadian Variations in Cortisol, Alpha Amylase, and Melatonin. *International Journal of Endocrinology* **2010**, *2010*, 829351, doi:10.1155/2010/829351.

57. Ivanova, I.A.; Danilenko, K.V.; Aftanas, L.I. Investigation of an Immediate Effect of Bright Light on Oxygen Consumption, Heart Rate, Cortisol, and α-Amylase in Seasonal Affective Disorder Subjects and Healthy Controls. *Neuropsychobiology* **2016**, *74*, 219-225, doi:10.1159/000477248.

9. Petrowski, K.; Buehrer, S.; Niedling, M.; Schmalbach, B. The effects of light exposure on the cortisol stress response in human males. *Stress* **2021**, *24*, 29-35, doi:10.1080/10253890.2020.1741543.

42. Petrowski, K.; Bührer, S.; Albus, C.; Schmalbach, B. Increase in cortisol concentration due to standardized bright and blue light exposure on saliva cortisol in the morning following sleep laboratory. *Stress* **2021**, *24*, 331-337, doi:10.1080/10253890.2020.1803265.

41. Petrowski, K.; Schmalbach, B.; Niedling, M.; Stalder, T. The effects of post-awakening light exposure on the cortisol awakening response in healthy male individuals. *Psychoneuroendocrinology* **2019**, *108*, 28-34, doi:10.1016/j.psyneuen.2019.05.016.

74. Sahin, L.; Wood, B.M.; Plitnick, B.; Figueiro, M.G. Daytime light exposure: Effects on biomarkers, measures of alertness, and performance. *Behav Brain Res* **2014**, *274*, 176-185, doi:10.1016/j.bbr.2014.08.017.

46. Schmidt, C.; Xhrouet, M.; Hamacher, M.; Delloye, E.; LeGoff, C.; Cavalier, E.; Collette, F.; Vandewalle, G. Light exposure via a head-mounted device suppresses melatonin and improves vigilant attention without affecting cortisol and comfort. *PsyCh Journal* **2018**, *7*, 163-175, doi:https://doi.org/10.1002/pchj.215.
